# Cultural Adaptation and Psychometric Evaluation of the Arabic Bernese Motive and Goal Inventory (Ar-BMZI) in Physical Health: A General Population Study Among Adults

**DOI:** 10.3390/healthcare14121750

**Published:** 2026-06-17

**Authors:** Nasser M. AbuDujain, Nawwaf N. Alharbi, Omar S. Alobaysi, Ariam M. Almsari, Mohammed K. Alqifari, Joud S. Almutairi, Khalid F. Alsadhan, Turky H. Almigbal, Abdulaziz Z. Alomar

**Affiliations:** 1 Department of Family and Community Medicine, College of Medicine, King Saud University, Riyadh P.O. Box 11495, Saudi Arabia; nasserabudujain@gmail.com (N.M.A.); dr.jouds@hotmail.com (J.S.A.); dr.khalidfm@gmail.com (K.F.A.); 2 College of Medicine, King Saud University, Riyadh P.O. Box 11495, Saudi Arabia; nawwaf.n.alharbi@gmail.com (N.N.A.); omar.s.alobaysi@gmail.com (O.S.A.); aryam.med443@gmail.com (A.M.A.); oamiq2001@gmail.com (M.K.A.); 3 Department of Orthopedic Surgery, College of Medicine, King Saud University, Riyadh P.O. Box 11495, Saudi Arabia

**Keywords:** BMZI, Bernese motive, exercise motive, validation, Saudi Arabia

## Abstract

**Background/aim**: Physical inactivity remains widespread globally, with most adults not achieving recommended physical activity levels. Exercise motives and goals, central to Self-Determination Theory, strongly influence sustained participation. The Bernese Motive and Goal Inventory (BMZI) is a validated tool to assess exercise motivation; however, no Arabic version exists. This study aimed to translate, culturally adapt, and validate the BMZI for Arabic-speaking adults. **Methods**: A web-based cross-sectional study was conducted in Saudi Arabia between September and October 2025 among native Arabic-speaking adults via social media and community networks. The survey included sociodemographic data, the Arabic version of the Bernese Motive and Goal Inventory (Ar-BMZI), the Sport Motivation Scale (SMS), and the SF-12 Health Survey. Reliability was assessed through Cronbach’s α, McDonald’s ω, and ICC for test–retest consistency; construct validity via exploratory and confirmatory factor analysis; and convergent validity by correlating Ar-BMZI with the Arabic-SMS and Arabic-SF-12 physical component. **Results**: A total of 680 participants were included, with a mean age of 30.4 ± 12.9 years. Most were female (61.6%) and held a bachelor’s degree (73.5%). Nearly half (50.9%) reported a low monthly income. The Ar-BMZI demonstrated strong overall psychometric performance. Internal consistency was excellent (Cronbach’s α = 0.883; ω = 0.868), and test–retest reliability indicated high stability over time (ICC = 0.870, 95% CI = 0.786–0.933). Convergent validity was supported by a moderate correlation with the Arabic Sport Motivation Scale (r = 0.613, *p* < 0.001) and a weak correlation with the SF-12 physical health domain (r = 0.098, *p* = 0.011), which supported discriminant validity. Exploratory principal component analysis with Varimax rotation identified a five-factor structure explaining 69.2% of the total variance, and confirmatory factor analysis further supported this structure, demonstrating an excellent model fit. **Conclusions**: The Ar-BMZI demonstrates high reliability and good validity, supporting its use among Arabic-speaking adults.

## 1. Introduction

Physical inactivity is a major global public health concern and a leading contributor to chronic disease, premature mortality, and rising healthcare costs. Saudi Arabia is particularly affected, with national data indicating that only 17.4% of adults meet recommended physical activity levels, leaving over 80% insufficiently active [1]. Similar findings have been reported in large population surveys, with low engagement in moderate and vigorous physical activity and high levels of sedentary behavior [2,3]. This challenge extends across the Arab region, where substantial variability in reported activity levels reflects differences in measurement approaches and highlights the need for standardized, culturally appropriate assessment tools [4]. Beyond physical health, inactivity contributes significantly to mortality, disability-adjusted life years (DALYs), and economic burden, with projections suggesting that improving activity levels in Saudi Arabia could prevent tens of thousands of deaths and yield substantial cost savings [5].

Importantly, physical inactivity is influenced not only by environmental and structural barriers but also by psychological and sociocultural factors. In Saudi Arabia, cultural norms, gender roles, and perceived barriers have been shown to significantly influence exercise behavior, with lack of motivation reported as a common reason for inactivity [6]. These findings underscore the importance of understanding the psychological determinants of physical activity, particularly motivation and goal orientation, within specific cultural contexts.

Self-Determination Theory (SDT) provides a widely accepted framework for understanding exercise motivation, distinguishing between autonomous motivation (e.g., exercising for enjoyment or personal value) and controlled motivation (e.g., exercising due to pressure or obligation) [7]. Autonomous forms of motivation have consistently been associated with greater adherence and sustained physical activity [8,9]. However, SDT primarily addresses how behavior is regulated rather than the specific goals individuals seek to achieve. Goal content theory, an extension of SDT, complements this perspective by focusing on the content of motivation, distinguishing intrinsic goals (e.g., health, personal growth) from extrinsic goals (e.g., appearance, social recognition) [10,11]. Together, these frameworks highlight the need for multidimensional tools that capture both the nature and content of exercise motivation.

Several instruments have been developed to assess exercise motivation, including the Physical Activity and Leisure Motivation Scale (PALMS) and the Exercise Motivations Inventory-2 (EMI-2). While widely used, these tools have demonstrated limitations in cross-cultural applications, often requiring structural modifications when adapted to different populations [12,13]. In addition, neither instrument is explicitly grounded in goal content theory, and no validated Arabic versions currently exist. These limitations highlight the need for culturally appropriate instruments with strong theoretical foundations.

**Figure 1:**
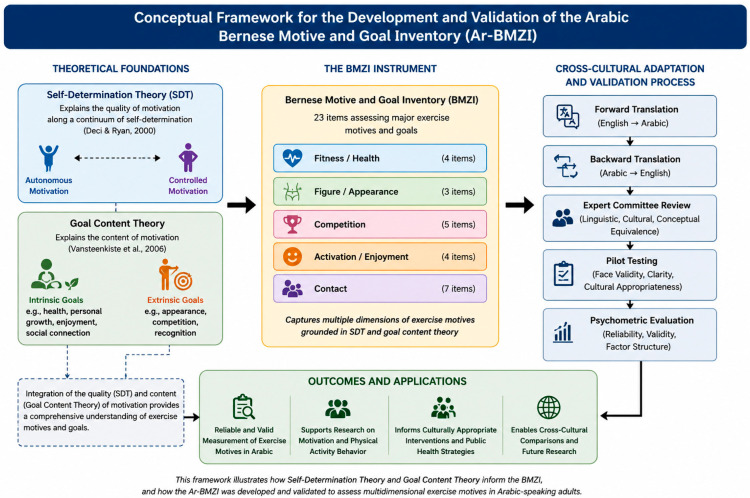
Conceptual framework for the development and validation of the Arabic Bernese Motive and Goal Inventory (Ar-BMZI). The framework illustrates the integration of Self-Determination Theory and goal content theory that underlie the BMZI, the cross-cultural adaptation process, and their applications in research and public health [7,10]. Figure generated using an AI-assisted visualization tool and refined by the authors.

The Bernese Motive and Goal Inventory (BMZI) was developed to address these gaps. Grounded in goal content theory, the BMZI assesses the specific motives and goals individuals associate with exercise rather than the regulatory processes underlying behavior [14,15]. The updated version comprises 23 items across multiple motivational domains and has demonstrated strong psychometric properties, including robust factor structure and reliability across independent samples [15]. Compared with other instruments, the BMZI offers a more concise and theoretically coherent framework for assessing exercise motivation. The conceptual framework underlying the development and validation of the Ar-BMZI is illustrated in Figure 1.

Despite its strengths, the BMZI has not been translated or validated in Arabic-speaking populations. Given the high prevalence of physical inactivity in Saudi Arabia and the broader Arab region, and the influence of cultural factors on exercise behavior, the absence of a validated Arabic instrument represents a significant gap. Therefore, this study aimed to translate, culturally adapt, and psychometrically evaluate the Arabic version of the BMZI (Ar-BMZI) among adults in Saudi Arabia. By providing a culturally appropriate and theoretically grounded tool, this study advances research on exercise motivation and supports the development of targeted public health interventions for Arabic-speaking populations.

## 2. Methodology

### 2.1. Study Characteristics

This study employed a cross-sectional design in Saudi Arabia from September 2025 to October 2025. We recruited participants in Saudi Arabia via social media platforms and community networks to maximize reach, using convenience sampling. A web-based, self-administered survey via the SurveyMonkey platform (www.surveymonkey.com). The survey composed five components: sociodemographic (age, gender, level of education, and monthly income), stage of behavior change, the Ar-BMZI, the Sport Motivation Scale (SMS-28), and the Short Form Health Survey (SF-12). To ensure methodological transparency for the web-based survey design, the study adhered to the Checklist for Reporting Results of Internet E-Surveys (CHERRIES) [16].

### 2.2. Participants

Eligibility criteria included: being a native Arabic speaker, currently residing in Saudi Arabia, and aged 18 years or older. Those who did not complete the survey were excluded. Sample size was calculated following Wolf et al.’s review to initially recruit a minimum of 10–20 observers per item, yielding a minimum of 230–460 participants [17]. For the face validation, a sample of 27 participants from the general adult population was recruited. The face validation phase targeted individuals holding at least a bachelor’s degree, as they were considered best positioned to provide accurate and meaningful feedback on the linguistic clarity, grammatical accuracy, and cultural appropriateness of the translated items. This educational criterion naturally resulted in a sample aged 30 to 65 years. The main validation study, however, included all native Arabic-speaking adults aged 18 years and above. Incomplete responses were excluded from the analysis; no imputation was performed.

### 2.3. Instruments

#### 2.3.1. Bernese Motive and Goal Inventory

The BMZI is a self-administered tool originally developed by Katrin Lehnert and colleagues in 2011 [14], subsequently updated by Schmid and colleagues in 2018 [15]. The original version comprised 24 items measuring motives across seven subscales: Contact, Competition/Performance, Activation/Enjoyment, Distraction/Catharsis, Figure/Appearance, Fitness/Health, and Aesthetics. In the updated version, the Activation/Enjoyment subscale and one Competition/Performance item were removed due to poor psychometric properties, and three new items were added to distinguish between Fitness and Health as separate constructs. The resulting updated BMZI consists of 23 items covering the following seven subscales: (1) Contact (4 items; socializing and maintaining social relationships through exercise), (2) Competition/Performance (3 items; comparing oneself with others and pursuing sport-related goals), (3) Distraction/Catharsis (4 items; using exercise to relieve stress and dispel negative emotions), (4) Body/Appearance (3 items; improving body weight and physical appearance), (5) Aesthetics (2 items; experiencing beautiful and harmonious movements during exercise), (6) Fitness (3 items; improving and maintaining physical performance and fitness), and (7) Health (3 items; improving and maintaining physical health). Each item is rated on a 5-point Likert scale (1 = ‘I strongly disagree’ to 5 = ‘I strongly agree’).

#### 2.3.2. Sport Motivation Scale

The Sport Motivation Scale (SMS) was initially developed in French by Brière et al. in 1995 [18]. SMS is a scale to measure motivation in the sport context. It consists of 28 items across seven subscales. Each of which includes four items. The scale assesses the three types of motivations: Intrinsic Motivation, Extrinsic Motivation, and Amotivation. The SMS-28 scale answered the question of “Why do you practice your sport?”. The answers to the scale’s question were rated on a seven-point Likert-type scale from (1—Does not correspond at all) to (7—corresponds exactly), with a midpoint of (4—corresponds moderately). We used the Arabic version of the SMS-28, which was psychometrically validated by Bayyat et al. in 2016, and has good psychometric properties [19].

#### 2.3.3. Short Form Health Survey

The SF-12 Health Survey is a 12-item instrument derived from the SF-36 [20], designed to measure health-related quality of life across key domains, including physical functioning, role limitations, bodily pain, general health perception, vitality, social functioning, and mental health. It yields two composite scores: the Physical Component Summary (PCS) and the Mental Component Summary (MCS). The SF-12 has demonstrated strong psychometric properties, with test–retest reliability coefficients typically exceeding 0.80 and Cronbach’s alpha values above 0.70 for both components. We used the Arabic version of SF-12, which was psychometrically validated by Haddad et al. In 2021, it had good psychometric properties [21].

### 2.4. Translation Procedure and Harmonization

The translation followed a rigorous process in accordance with established guidelines [22,23]. Initially, two independent professional translators performed the forward translation, one of whom held a medical degree. The two versions were then compared and merged by the authors, N.M.A. and A.Z.A., resulting in the preliminary reconciled forward version. This version was then sent for backward translation into English by a separate translator who was not involved in the forward translation process and had no prior knowledge of the original instrument.

To ensure the backward translation conveyed the intended meaning as the original, we presented both the backward English version and the original English to three medical professionals with a strong background; furthermore, a third native German speaker, to compare with the primarily German-developed version. This step was undertaken to ensure conceptual equivalence with the original German-developed instrument. The items “*For enjoyment of beautiful movements in exercise*” and “*To make new friends through exercise*” seemed to have partially lost their meaning during translation.

The polished version was then subjected to a face validation process involving 27 participants from the general population aged 30 to 65 years. Participants raised several comments during face validation, mainly about grammar and syntax. Feedback was evaluated qualitatively. After this process, the authors N.M.A., N.A., and O.S.A. held a meeting to discuss the appropriateness of the comments and began implementation accordingly. Further revision was done to ensure accuracy in the text transference, which eventually resulted in the final Ar-BMZI available in the Supplementary File S1.

### 2.5. Ethical Considerations

Ethical approval for this study was obtained from the Institutional Review Board of King Saud University (No. E-25-10051; 25/0657/IRB) on 26 August 2025. Given the web-based nature of the survey, ethical considerations were maintained through the following procedural measures: (1) Informed consent: the first page of the online survey presented a clear description of the study purpose, procedures, potential benefits, and voluntary nature of participation. Participants were required to actively click an ‘I agree’ button to proceed, constituting explicit electronic informed consent. (2) Confidentiality and anonymity: no personally identifiable information (such as name, national ID, or IP address) was collected. All data were stored on password-protected servers accessible only to the research team. (3) Right to withdraw: participants were informed that they could close the browser at any time without submitting partial responses, with no penalty for non-completion.

### 2.6. Statistical Analysis

Data were analyzed using IBM SPSS Statistics, version 31.0 (IBM Corp., Armonk, NY, USA). Descriptive statistics were used to summarize participants’ sociodemographic characteristics, presented as means and standard deviations (SD) for continuous variables and frequencies and percentages for categorical variables. Reliability was examined using Cronbach’s alpha, alpha based on standardized items, and McDonald’s omega coefficients, with values ≥0.70 considered acceptable. Test–retest reliability was evaluated using a two-way mixed-effects intraclass correlation coefficient (ICC) with absolute-agreement ICCs and 95% CIs in 28 participants over 14 days. Agreement between test and retest scores was visualized using a Bland–Altman plot to detect potential systematic bias. Construct validity was examined using principal component analysis (PCA), with the Kaiser–Meyer–Olkin (KMO) statistic and Bartlett’s test of sphericity confirming sampling adequacy and suitability of correlations. PCA was selected as an exploratory technique to examine the dimensional structure of the Arabic BMZI and identify patterns of item clustering within the translated instrument. This approach has been widely used in questionnaire validation studies during the exploratory phase and was followed by confirmatory factor analysis (CFA) to evaluate the stability and adequacy of the resulting factor structure. Factor retention was guided by eigenvalues > 1 and visual inspection of the scree plot. Item-level assumptions for all 23 items were assessed using the KMO statistic. Univariate normality was evaluated for each item through skewness and kurtosis indices.

To further validate exploratory analysis, we performed a confirmatory factor analysis (CFA) using RStudio (R version 4.5.1). CFA was conducted using weighted least squares with mean and variance adjustment (WLSMV) estimation, appropriate for ordinal Likert-scale responses. Model fit was evaluated using multiple fit indices: Comparative Fit Index (CFI), Tucker–Lewis Index (TLI), Root Mean Square Error of Approximation (RMSEA) with 90% confidence intervals, and Standardized Root Mean Square Residual (SRMR). Models were considered to demonstrate excellent fit if CFI and TLI exceeded 0.95, RMSEA was less than 0.08 with the lower bound of the 90% CI below 0.05, and SRMR was less than 0.08. Standardized factor loadings and their *p*-values were examined to confirm that all items loaded significantly on their respective factors.

An exploratory receiver operating characteristic (ROC) analysis was additionally performed to evaluate the ability of the total Ar-BMZI score to discriminate between participants who were currently physically active and those who were not, based on the self-reported stage-of-change classification. Participants in the action and maintenance stages were classified as physically active (positive outcome), whereas those in the precontemplation, contemplation, and preparation stages were classified as not currently physically active (negative outcome). Given the absence of an established diagnostic application for the BMZI, this analysis was considered exploratory and hypothesis-generating.

Convergent validity was tested via Pearson’s correlation between the total Ar-BMZI score and conceptually related measures—the Physical Component Summary (PCS) of the SF-12 and the Arabic Sport Motivation Scale (Ar-SMS). All tests were two-tailed, and *p*-values < 0.05 were considered statistically significant.

### 2.7. Declaration of AI Use

Artificial intelligence (AI) tools were used to assist in generating a conceptual framework figure included in this manuscript. The content, interpretation, and final presentation were reviewed and validated by the authors. No AI tools were used for data analysis or primary scientific content generation.

## 3. Results

**Figure 2:**
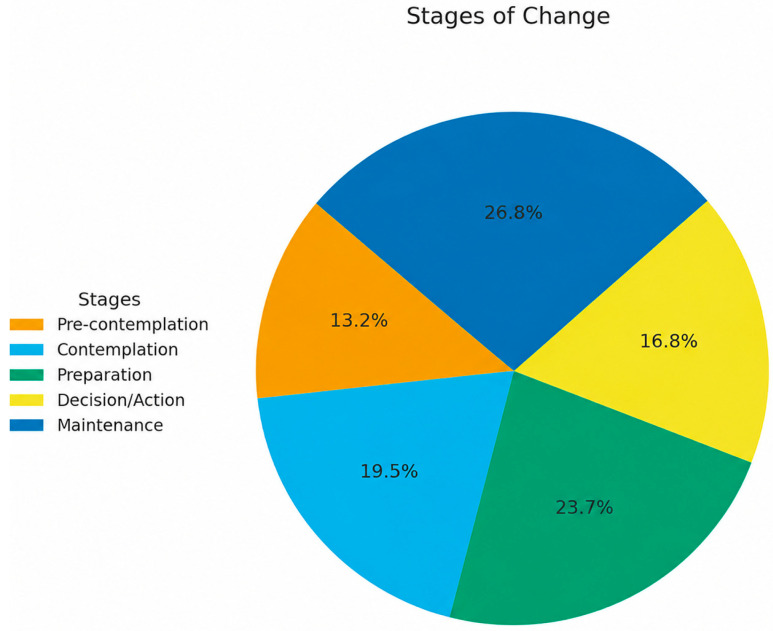
Distribution of participants across stages of behavior change. Participants were classified according to the Transtheoretical Model (TTM) into pre-contemplation, contemplation, preparation, action, and maintenance stages based on self-reported physical activity behavior at the time of assessment.

A total of 680 participants were included in the study, with a mean age of 30.41 ± 12.93 years, indicating a relatively young adult sample (Table 1). Females accounted for 61.6% of respondents, and males for 38.4%. Most participants held a bachelor’s degree (73.5%), 20.1% had a high school education or below, and 6.3% had postgraduate qualifications. Approximately half of the participants (50.9%) reported being in the lowest monthly income category, 30.3% in the middle-income range, 14.7% in the higher-middle bracket, and 4.1% in the highest income level. The distribution of stages of change is presented in Figure 2.

**Figure 3:**
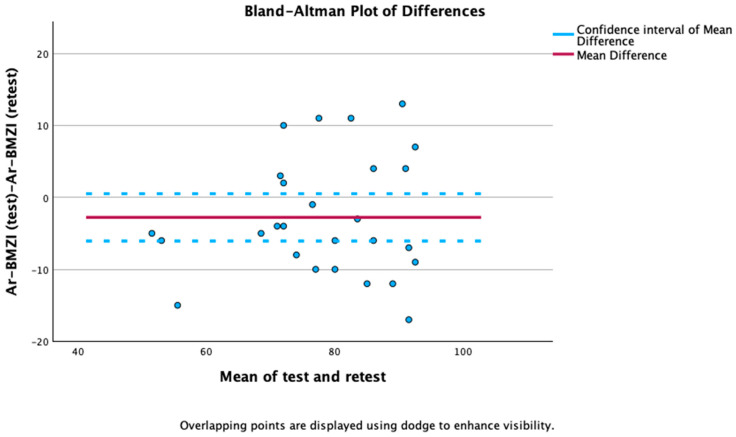
Bland–Altman plot of test–retest responses. Test–retest agreement was assessed in a subsample of 28 participants over a two-week interval, demonstrating good agreement with most observations within the 95% limits of agreement and no evidence of systematic bias.

Reliability testing indicated excellent internal consistency, with a Cronbach’s alpha of 0.883 and an alpha based on standardized items of 0.882 (Table 2). McDonald’s omega was 0.868, further confirming reliability. The mean total score was 81.21 ± 13.44 with a variance of 180.61, reflecting acceptable variability among participants. The data were suitable for factor analysis, as indicated by a meritorious KMO value (0.862) and a significant Bartlett’s test of sphericity (χ^2^ = 8965.67, df = 253, *p* < 0.001). Test–retest reliability, assessed via the intraclass correlation coefficient (ICC), indicated excellent stability over time (ICC = 0.870, 95% CI = 0.786–0.933, *p* < 0.001). The Bland–Altman plot (Figure 3) confirmed consistency between test and retest scores, with most observations within the 95% limits of agreement and with no systematic bias, supporting reproducibility.

### 3.1. Convergent Validity

The total Ar-BMZI score showed a moderate positive correlation with the Arabic Sport Motivation Scale (Ar-SMS) (r = 0.613, *p* < 0.001), consistent with convergence between two closely related motivational constructs and supporting the instrument’s convergent validity.

### 3.2. Discriminant Validity

**Figure 4:**
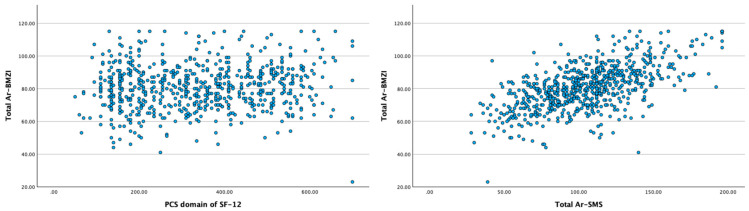
Scatterplots showing convergent and discriminant validity of the Arabic Bernese Motive and Goal Inventory (Ar-BMZI). Left: Relationship between total Ar-BMZI and the Physical Component Summary (PCS) of the SF-12 (r = 0.098, *p* = 0.011). Right: Relationship between total Ar-BMZI and the Arabic Sport Motivation Scale (Ar-SMS) (r = 0.613, *p* < 0.001). Each point represents an individual participant; solid clustering in the right panel illustrates a stronger linear association than in the left panel.

By contrast, the Ar-BMZI total score showed a weak but statistically significant correlation with the Physical Component Summary (PCS) of the SF-12 (r = 0.098, *p* = 0.011). This weak association is expected, as exercise motives represent proximal psychological drivers of behavior, whereas the SF-12 physical component reflects broader health burden including disease, pain, and functional limitations. As illustrated in Figure 4, this pattern supports the discriminant validity of the Ar-BMZI, indicating it measures a construct distinct from general physical health status.

All 23 items of the BMZI demonstrated satisfactory internal consistency and a substantial contribution to the scale’s overall reliability. The corrected item–total correlations ranged from 0.303 to 0.672. The “Cronbach’s alpha if item deleted” values ranged between 0.872 and 0.884. The scale mean if the item were deleted ranged from 77.3 to 78.8, with variances ranging from 160 to 174.

**Figure 5:**
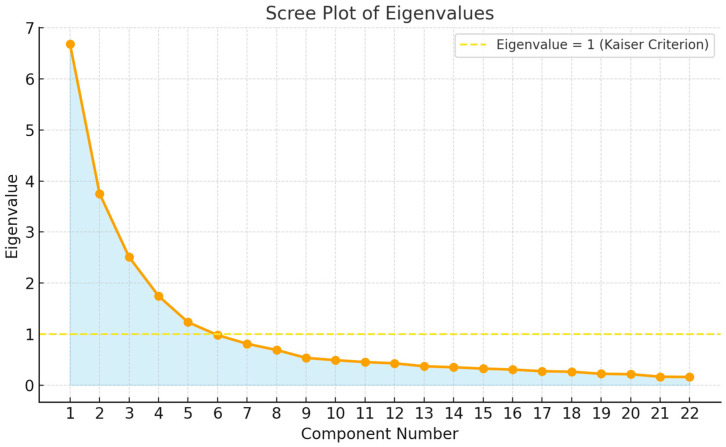
Scree plot of eigenvalues for the Arabic BMZI scale. The analysis extracted five components with eigenvalues greater than 1, accounting for 69.2% of the variance (Component 1 = 29.0%, Component 2 = 16.3%, Component 3 = 10.9%, Component 4 = 7.6%, and Component 5 = 5.4%). As shown in the plot, the eigenvalues sharply declined after the fifth component, forming an apparent “elbow” that supports the retention of a five-factor solution according to the Kaiser criterion (eigenvalue > 1). This finding confirms the multidimensional structure of the Arabic BMZI, consistent with the theoretical framework of the original instrument.

The Kaiser–Meyer–Olkin (KMO) measure of sampling adequacy was 0.862, exceeding the recommended threshold of 0.60. Bartlett’s test of sphericity was significant (χ^2^ = 8965.67, df = 253, *p* < 0.001). In the current study, the number of dimensions of the Arabic BMZI was determined using the Kaiser criterion (eigenvalue > 1.0), scree plot test, and cumulative variance explained by factors. Thus, although seven theoretical domains were proposed in the original BMZI, PCA of the data identified five empirical factors, accounting for a total variance of 69.2%. For transparency and comparison with the original structure, the factor loadings from the initial seven-factor solution are provided in Supplementary Table S1. Based on factor loadings, we combined two domains into a single factor: the original Fitness and Health domains were merged into a Fitness factor, while the original Competition and Aesthetics domains were combined into a Competition factor. These consolidations were supported by strong empirical evidence: Fitness and Health items showed a high inter-factor correlation (r = 0.824, *p* < 0.001), justifying their merger into a unified Fitness/Health factor, while Competition and Aesthetics items showed substantial overlap (r = 0.686, *p* < 0.001), supporting their combination into a single Competition/Performance and Aesthetics factor. Furthermore, the seven-factor CFA model revealed problematic cross-loadings inconsistent with the original theoretical structure, whereas the five-factor model demonstrated superior parsimony and interpretability, as confirmed by both PCA and CFA fit indices. Retaining the five-factor solution therefore provided the most parsimonious representation of the data while preserving conceptual coherence across motivational domains. The other three factors (Distraction, Contact, and Figure/Appearance) mapped onto the original structure perfectly (Table 3). The rotated component matrix demonstrated that items 1–4 loaded strongly on Component 3, items 5–9 on Component 2, items 11–14 on Component 4, items 17–20 on Component 1, and items 21–23 on Component 5. Each component showed a simple structure and minimal cross-loadings, confirming the presence of five distinct factors. These five components (Figure 5) corresponded conceptually to the following motivational domains: Contact, Fitness/Health, Distraction/Catharsis, Competition/Performance and Aesthetics, and Figure/Appearance.

Item-level Kaiser–Meyer–Olkin (KMO) values ranged from 0.72 to 0.94 (all ≥0.70, acceptable). Skewness values ranged from −1.90 to 0.46 (within the acceptable −2 to +2 range). Kurtosis values ranged from 1.97 to 8.10. While some items showed elevated kurtosis, this is typical for 5-point ordinal data and does not affect the validity of PCA with large sample sizes. Item-level details are presented in Table 3.

The five-factor model was then validated through confirmatory factor analysis. As shown in Table 4, all 23 items loaded significantly on their respective factors (standardized loadings λ = 0.61–0.98, all *p* < 0.001). The five-factor CFA model demonstrated an acceptable-to-excellent fit to the data: χ^2^(220) = 1512.50, *p* < 0.001, CFI = 0.991, TLI = 0.990, RMSEA = 0.091 [90% CI: 0.087–0.096], SRMR = 0.074. Although the RMSEA value was slightly above conventional thresholds, the CFI, TLI, and SRMR all indicated good-to-excellent model fit. Previous psychometric research has shown that RMSEA may be sensitive to sample size and model complexity and can occasionally suggest poorer fit despite favorable performance on other fit indices. Therefore, model adequacy was evaluated using multiple complementary indices rather than relying on a single statistic. Taken together, the results support the acceptability of the five-factor measurement structure. In addition, RMSEA is known to be relatively sensitive in large-sample CFA models using ordinal indicators and should be interpreted alongside other global fit indices rather than in isolation. For comparison, confirmatory factor analysis results for the original seven-factor model are presented in Supplementary Table S2.

Internal consistency was excellent across all factors. Cronbach’s alpha ranged from 0.81 to 0.94, and McDonald’s omega ranged from 0.86 to 0.98 (Table 4), both indicating good to excellent reliability. Average variance extracted (AVE) ranged from 0.71 to 0.89, supporting strong convergent validity.

Inter-factor correlations are presented in Table 5. Correlations among the five subscales ranged from 0.064 to 0.654, indicating that factors are related but conceptually distinct. The strongest correlation was between Competition and Contact (r = 0.654, *p* < 0.001). Fitness and Figure/Appearance showed a moderate correlation (r = 0.457, *p* < 0.001), and distraction showed the weakest overall correlation with other subscales, with no significant correlation with Figure/Appearance (r = 0.064, non-significant). An exploratory ROC analysis was performed to assess the ability of the total Ar-BMZI score to distinguish between participants who were currently physically active (action/maintenance stages) and those who were not (precontemplation, contemplation, or preparation stages). The analysis demonstrated weak but statistically significant discrimination (AUC = 0.591, 95% CI: 0.548–0.634, *p* < 0.001). Detailed results are provided in Supplementary Figure S1.

## 4. Discussion

This study translated, culturally adapted, and psychometrically evaluated the BMZI for Arabic-speaking adults in Saudi Arabia. The Ar-BMZI demonstrated a clear five-factor structure, strong internal consistency, good test–retest reliability, and expected patterns of association with related constructs, supporting its overall validity.

Exercise motivation is most commonly conceptualized through Self-Determination Theory (SDT), which emphasizes the role of motivation quality in promoting sustained physical activity [7]. SDT describes a continuum ranging from controlled motivation, driven by external pressures or obligations, to autonomous motivation, which reflects intrinsic enjoyment or personally valued goals [24,25]. Environments that support autonomy, competence, and relatedness are more likely to foster autonomous motivation and long-term engagement in physical activity [7]. Substantial empirical evidence supports this framework, with systematic reviews demonstrating that intrinsic and identified regulation are consistently associated with exercise adherence and maintenance, whereas controlled forms show weaker and less consistent associations [8,9]. Evidence from Arabic-speaking populations further supports the applicability of SDT, with studies showing that physical activity levels are positively associated with perceived competence and autonomous motivation, and that gender differences in motivation may partly explain disparities in participation [26].

However, while SDT explains how behavior is regulated, it provides limited insight into the specific goals individuals seek to achieve through exercise. Goal content theory extends this perspective by focusing on the substance of motivation, distinguishing intrinsic goals—such as health, personal growth, and mastery—from extrinsic goals—such as appearance and social recognition [10,11]. This distinction is important, as individuals with similar levels of motivation may differ substantially in their underlying goals and associated outcomes. Intrinsic goal content has been consistently linked to greater well-being, need satisfaction, and sustained physical activity, whereas extrinsic goals are often associated with less favorable psychological outcomes [11]. Together, these frameworks highlight that a comprehensive understanding of exercise motivation requires assessing both regulatory processes and goal content, an approach reflected in multidimensional instruments such as the BMZI, which captures the specific motives underlying exercise behavior.

Beyond aligning with self-determination and goal content theory, our findings are also consistent with SDT-based motivation research from non-Western settings. For example, the PALMS has demonstrated stable, SDT-consistent factor structures in Malaysian and Chinese populations, with domains such as appearance, health, enjoyment, and social motives consistently identified [12,27]. These cross-cultural replications suggest that core exercise motivation constructs may be broadly generalizable, supporting the interpretation that the Ar-BMZI reflects fundamental motivational dimensions rather than culturally specific patterns.

Validated instruments such as the PALMS and the EMI-2 are widely used to assess exercise motivation in population-based research. PALMS offers a relatively concise multidimensional assessment and has demonstrated acceptable reliability in its original validation; however, its factorial structure has shown instability across cultural adaptations, requiring modifications in several language versions [12,28,29,30]. Similarly, the EMI-2 provides a comprehensive but complex framework with multiple factors, yet its structure has proven difficult to replicate consistently, with cross-cultural studies frequently reporting reduced or reorganized factor solutions [13,31,32,33]. Beyond these structural inconsistencies, both instruments lack a clear grounding in goal content theory and have not been formally validated in Arabic-speaking populations, limiting their applicability in this context. These limitations highlight the need for a theoretically coherent and culturally adaptable instrument, which informed the selection of the BMZI, a measure explicitly grounded in goal content theory and designed to capture the multidimensional motives underlying exercise behavior.

Exploratory principal component analysis with Varimax rotation yielded five meaningful factors—Contact, Fitness/Health, Distraction/Catharsis, Competition/Performance and Aesthetics, and Figure/Appearance—that together explained about two-thirds of the total variance. This factor configuration maintains the essential BMZI domains while merging closely related content into broader factors, which is consistent with the original framework [14]. Compared with the updated 23-item BMZI, which reorganizes the original domains into a slightly different set of scales and no longer includes a distinct Activation/Enjoyment dimension, our five-factor solution provides a more parsimonious structure while preserving core motivational constructs, a pattern that was further supported by confirmatory factor analysis demonstrating acceptable model fit [15]. By showing that a BMZI-based structure also fits an Arabic-speaking, non-Western community sample and aligns with reports that appearance- and health-related motives tend to cluster in community-based studies across different regions [15,34,35,36], this study adds to the cross-cultural support for the BMZI’s fundamental motive domains.

The observed consolidation of the Fitness and Health domains may reflect the tendency to view these concepts as closely intertwined in community-based Arabic-speaking populations, where improving physical fitness is often regarded as integral to maintaining general health. Likewise, the overlap between Competition/Performance and Aesthetics may indicate that achievement-oriented motives and appearance-related aspirations are not perceived as entirely distinct goals, particularly outside elite athletic settings. Similar modifications of the original factor structure have been reported during cross-cultural adaptation of other exercise motivation instruments, suggesting that these differences may reflect cultural interpretation rather than a loss of construct validity.

The five-factor solution demonstrated strong psychometric properties across both its internal structure and reliability. KMO values, together with Bartlett’s test, indicated that the correlation matrix was appropriate for factor analysis, and standard factor retention rules converged on the same five-factor pattern. The total-scale reliability estimate was high, and item-level checks indicated that all items contributed meaningfully to their respective factors, with no item that would have improved reliability if removed (Table 2). After rotation, items loaded strongly on their assigned factors, with minimal overlap across other factors, suggesting that each factor represented a distinct dimension of exercise motivation in this group. These findings were reinforced by confirmatory factor analysis, which supported the structural validity of the five-factor model and indicated an acceptable-to-good fit across multiple indices.

The present findings should also be interpreted in light of the theoretical and developmental characteristics of the BMZI. Unlike widely used instruments such as PALMS and EMI-2, which primarily emerged from empirical item pools, the BMZI is explicitly grounded in goal content theory and was designed to assess the substance of exercise motivation rather than its regulatory processes [14,15]. This theoretical grounding may explain the coherent and interpretable factor structure observed in the current study despite cultural adaptation. In addition, the relatively concise structure of the BMZI (23 items) likely contributed to the high internal consistency observed, while maintaining coverage of key motivational domains relevant to population-based research. Its demonstrated adaptability across age groups and settings further supports its suitability for cross-cultural application, and the absence of prior validation highlights the contribution of the present study.

Construct validity was supported by the expected pattern of associations with external measures (Figure 4). The Ar-BMZI total score correlated moderately to strongly with the Arabic Sport Motivation Scale, consistent with convergence between two closely related motivational constructs. By contrast, its association with the SF-12 physical component summary was small, which aligns with the view that exercise motives are proximal, domain-specific drivers of behavior, whereas the SF-12 physical health component reflects broader influences such as comorbidities, pain, and functional limitations. This supports discriminant validity, indicating that exercise motives are distinct from general physical health status.

This pattern accords with the role of motives as proximal drivers of sport and exercise rather than as proxies for global physical health and is consistent with prior work on motives inventories and the BMZI program more broadly [14,15,34,35,36]. In this study, evidence for construct validity is primarily based on correlations with external measures and an exploratory ROC analysis evaluating the ability of the total Ar-BMZI score to distinguish between participants who were currently physically active and those who were not, according to their self-reported stage of behavior change. However, given the relatively low AUC, these findings should be interpreted cautiously and do not support the use of the instrument as a diagnostic or screening tool.

This study highlights several methodological and theoretical strengths. Rigorous forward–backward translation and expert review ensured conceptual equivalence, clarity, and precision of the Arabic BMZI. The instrument has strong psychometric properties and offers meaningful implications for both research and practice in sport and exercise psychology. However, several limitations should be considered when interpreting the findings. One limitation of our study is that we recruited participants through online convenience sampling via social media, which may have introduced selection bias. First, there were more females (61.6%) and highly educated individuals (73.5% had a bachelor’s degree) than would be seen in a probability-based sample of the general Saudi adult population. This demographic profile likely reflects the characteristics of voluntary web-based surveys in Saudi Arabia and should be considered when interpreting the external validity and generalizability of the findings. It is also possible that females and highly educated participants have different exercise motivation profiles than males and less educated adults, which would limit the generalizability of this specific factor structure and subscale scores to these groups. Second, by recruiting online, we may have overrepresented younger adults who are more digitally engaged and likely underrepresented older adults and those who may not have had access to social media or the internet, such as rural adults. Thus, our results may generalize to digitally accessible adults and not necessarily all adults in Saudi Arabia or adults who speak Arabic as their first language. Probability-based samples are needed to replicate these results.

Additionally, the cross-sectional, self-report design allows assessment of associations at a single time point, but it does not capture temporal changes or causal relationships and may be prone to common-method bias. While the Ar-BMZI showed strong psychometric properties, it is not intended as a diagnostic or screening tool and may lack sensitivity or specificity for clinical use. The Ar-BMZI is validated for assessing exercise motives, not for diagnostic or behavior-change screening purposes. Finally, the instrument was culturally adapted in Saudi Arabia, and further validation is needed in diverse Arabic-speaking populations, as exercise motives may vary across regions. The Arabic BMZI provides a standardized, culturally appropriate tool for assessing exercise and sport motives and goals among Arabic-speaking adults. It has potential applications in public health surveillance to understand motivational determinants of physical inactivity, as well as in clinical, counseling, community, and rehabilitation settings to tailor exercise interventions according to individual motives. Moreover, it enables population segmentation based on motivational profiles, thereby guiding targeted health promotion campaigns that align with cultural values and personal goals.

Future studies should test longitudinal invariance, domain-level validity, and responsiveness to interventions. Further research using probability-based sampling and more diverse population groups is warranted to enhance external validity. Theoretically, the Arabic BMZI extends the application of self-determination theory and goal content theory to Arabic-speaking contexts, thereby advancing motivation-based physical activity research in the region.

## 5. Conclusions

This study translated and validated the Arabic version of the Ar-BMZI among adults in Saudi Arabia. The findings support a five-factor structure with good reliability and acceptable construct validity, supported by both exploratory and confirmatory analyses. The Ar-BMZI provides a culturally appropriate and theoretically grounded tool for assessing exercise motivation in Arabic-speaking populations, with potential applications in research and public health. However, the use of web-based sampling and the cross-sectional design may limit generalizability. The observed five-factor structure should be interpreted as a culturally adapted representation of the original BMZI framework and warrants further evaluation in other Arabic-speaking populations. Further studies are needed to confirm these findings across diverse Arabic populations and to examine longitudinal stability and responsiveness to interventions.

## Figures and Tables

**Table 1:** Sociodemographic Characteristics of the Study Participants (n = 680).

Variable		n	(%)
Age	Mean ± SD	30.41 ± 12.929	
Gender	Male	261	38.4
	Female	419	61.6
Level of education	High school and below	137	20.1
	Bachelor’s degree	500	73.5
	Postgraduate degree	43	6.3
Monthly income	Less than 5000 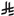	346	50.9
	from 5000 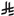 to less than 15,000 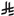	206	30.3
	from 15,000 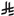 to less than 30,000 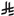	100	14.7
	More than 30,000 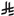	28	4.1

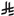
 = 0.27 United States Dollar.

**Table 2:** Item Analysis and Reliability Coefficients for the Arabic BMZI Scale.

	Scale Mean if Item Deleted	Scale Variance if Item Deleted	Corrected Item-Total Correlation	Squared Multiple Correlation	Cronbach’s Alpha if Item Deleted
BMZI 1	77.55	168.242	0.403	0.586	0.880
BMZI 2	77.80	167.562	0.386	0.544	0.881
BMZI 3	77.53	167.507	0.427	0.647	0.880
BMZI 4	77.58	167.006	0.438	0.510	0.880
BMZI 5	76.67	174.249	0.328	0.533	0.882
BMZI 6	76.82	172.903	0.341	0.627	0.882
BMZI 7	76.81	173.194	0.349	0.646	0.882
BMZI 8	76.70	172.518	0.400	0.596	0.881
BMZI 9	76.90	171.209	0.362	0.503	0.881
BMZI 10	77.34	167.830	0.379	0.306	0.881
BMZI 11	77.96	160.934	0.586	0.502	0.875
BMZI 12	78.65	163.265	0.499	0.468	0.878
BMZI 13	77.46	165.533	0.514	0.331	0.877
BMZI 14	78.01	160.921	0.559	0.713	0.876
BMZI 15	78.11	159.854	0.601	0.719	0.875
BMZI 16	78.48	158.644	0.652	0.708	0.873
BMZI 17	78.54	157.999	0.672	0.761	0.872
BMZI 18	78.59	159.744	0.617	0.764	0.874
BMZI 19	78.75	160.259	0.607	0.866	0.874
BMZI 20	78.76	160.616	0.592	0.828	0.875
BMZI 21	77.27	169.480	0.308	0.614	0.884
BMZI 22	77.00	171.315	0.330	0.585	0.882
BMZI 23	77.36	170.103	0.303	0.459	0.883
Total 23 items	Scale mean	SD	Variance	Cronbach’s α	McDonald’s ω
	81.21	13.439	180.614	0.883	0.868

**Table 3:** Rotated Component Matrix Showing Factor Loadings of the Arabic BMZI (Principal Component Analysis with Varimax Rotation).

	Factor				
	1 (Contact)	2 (Fitness/Health)	3 (Distraction/Catharsis)	4 (Competition/Performance and Aesthetics)	5 (Figure/Appearance)
BMZI 1	0.067	0.075	**0.858**	0.108	0.007
BMZI 2	0.126	−0.050	**0.822**	0.120	0.041
BMZI 3	0.089	0.055	**0.880**	0.109	0.039
BMZI 4	0.108	0.147	**0.772**	0.184	−0.057
BMZI 5	−0.083	**0.788**	0.124	0.102	0.051
BMZI 6	−0.039	**0.827**	0.002	0.138	0.069
BMZI 7	−0.009	**0.849**	−0.011	0.146	0.005
BMZI 8	0.048	**0.809**	0.035	0.061	0.184
BMZI 9	0.098	**0.694**	0.056	−0.043	0.261
BMZI 10	0.267	**0.486**	0.060	0.001	0.192
BMZI 11	0.376	0.124	0.234	**0.540**	0.059
BMZI 12	0.433	−0.006	0.146	**0.471**	0.060
BMZI 13	0.242	0.287	0.088	**0.472**	0.187
BMZI 14	0.215	0.093	0.126	**0.862**	−0.005
BMZI 15	0.256	0.079	0.180	**0.828**	0.034
BMZI 16	**0.769**	0.025	0.090	0.370	0.076
BMZI 17	**0.825**	0.045	0.124	0.302	0.062
BMZI 18	**0.882**	0.045	0.096	0.185	−0.004
BMZI 19	**0.922**	0.024	0.068	0.130	0.033
BMZI 20	**0.901**	0.040	0.063	0.125	0.022
BMZI 21	0.050	0.127	0.037	0.039	**0.891**
BMZI 22	0.048	0.223	−0.007	0.050	**0.844**
BMZI 23	0.033	0.194	−0.011	0.083	**0.791**

**Table 4:** Confirmatory factor analysis results for standardized factor loadings and reliability estimates for the five-factor Ar-BMZI model.

Factor	Factor Loadings			Reliability		
	Item	Standardized Values	*p*-Value	Alpha	McDonald Omega	AVE
Competition/Performance and Aesthetics	BMZI_11	0.752		0.806	0.971	0.708
	BMZI_12	0.704	<0.001			
	BMZI_13	0.613	<0.001			
	BMZI_14	0.893	<0.001			
	BMZI_15	0.917	<0.001			
Contact	BMZI_16	0.876		0.942	0.979	0.894
	BMZI_17	0.908	<0.001			
	BMZI_18	0.902	<0.001			
	BMZI_19	0.977	<0.001			
	BMZI_20	0.947	<0.001			
Distraction/Catharsis	BMZI_1	0.84		0.873	0.879	0.715
	BMZI_2	0.814	<0.001			
	BMZI_3	0.893	<0.001			
	BMZI_4	0.797	<0.001			
Figure/Appearance	BMZI_21	0.879		0.836	0.855	0.775
	BMZI_22	0.917	<0.001			
	BMZI_23	0.79	<0.001			
Fitness/Health	BMZI_10	0.607	<0.001	0.833	0.905	0.722
	BMZI_5	0.836				
	BMZI_6	0.889	<0.001			
	BMZI_7	0.897	<0.001			
	BMZI_8	0.875	<0.001			
	BMZI_9	0.780	<0.001			

Fit indicators: χ^2^(220) = 1512.5, *p* < 0.001, CFI = 0.991, TLI = 0.99, RMSEA = 0.091 (90% CI, 0.087 to 0.096), and SRMR = 0.074.

**Table 5:** Standardized correlations between Ar-BMZI factors (five factors). r = correlation coefficient.

Factor 1	Factor 2	r	*p*-Value
Distraction	Fitness	0.172	<0.001
	Competition	0.442	<0.001
	Contact	0.292	<0.001
	Figure	0.064	0.163
Fitness	Competition	0.334	<0.001
	Contact	0.132	0.003
	Figure	0.457	<0.001
Competition	Contact	0.654	<0.001
	Figure	0.208	<0.001
Contact	Figure	0.125	0.004

## Data Availability

Data are available upon request from the corresponding author due to privacy reasons.

## Supplementary Materials

The following supporting information can be downloaded at: https://www.mdpi.com/article/10.3390/healthcare14121750/s1. File S1: Bernese Motive and Goal Inventory (BMZI). File S2: Table S1: Factor Loadings of the seven-factor solution of the Arabic BMZI (Principal Component Analysis With Varimax Rotation). Table S2: Confirmatory factor analysis results for standardized factor loadings and reliability estimates for the seven-factor Ar-BMZI model. Table S3: Standardized correlations between Ar-BMZI factors (seven factors). Figure S1: For the Exploratory ROC curve analysis, participants in the action and maintenance stages of change were classified as the positive group, whereas those in pre-contemplation, contemplation, and preparation were classified as the negative group. File S3: Checklist for Reporting Results of Internet E-Surveys (CHERRIES).

## Abbreviations

Ar-BMZI	Arabic Bernese Motive and Goal Inventory
AUC	Area Under the Curve
BMZI	Bernese Motive and Goal Inventory
CI	Confidence Interval
CFA	Confirmatory Factor Analysis
EFA	Exploratory Factor Analysis
ICC	Intraclass Correlation Coefficient
IRB	Institutional Review Board
KMO	Kaiser–Meyer–Olkin (Measure of Sampling Adequacy)
MCS	Mental Component Summary (SF-12)
PCS	Physical Component Summary (SF-12)
PCA	Principal Component Analysis
ROC	Receiver Operating Characteristic
SD	Standard Deviation
SDT	Self-Determination Theory
SF-12	12-Item Short Form Health Survey
SMS	Sport Motivation Scale
SMS-28	Sport Motivation Scale, 28-item version
ω (Omega)	McDonald’s Omega Reliability Coefficient
α (Alpha)	Cronbach’s Alpha Reliability Coefficient
χ^2^	Chi-square Statistic
