# Supplementary material for: Cultural Adaptation and Psychometric Evaluation of the Arabic Bernese Motive and Goal Inventory (Ar-BMZI) in Physical Health: A General Population Study Among Adults
Source: Healthcare (Basel). 2026 Jun 17;14(12):1750. doi: 10.3390/healthcare14121750 (PMC13299709; doi:10.3390/healthcare14121750)
Supplement: Supplementary file 1 [file healthcare-14-01750-s001.zip › healthcare-4282399-supplementary file S2.pdf]

Table S1: Factor Loadings of the seven-factor solution of the Arabic BMZI (Principal Component Analysis With Varimax Rotation)

Variable	Factors							Assumptions		
	RC1	RC2	RC3	RC4	RC5	RC6	RC7	KMO[1]	Skewness[1]	Kurtosis[1]
BMZI_1	0.069	0.056	**0.862**	0.007	0.104	0.015	0.046	0.863	-0.74	3.033
BMZI_2	0.123	-0.05	**0.824**	0.041	0.079	0.083	-0.02	0.868	-0.46	2.318
BMZI_3	0.082	0.046	**0.880**	0.041	0.059	0.102	0.022	0.819	-0.79	3.022
BMZI_4	0.107	0.135	**0.775**	-0.05	0.148	0.095	0.052	0.898	-0.67	2.776
BMZI_5	-0.03	**0.821**	0.138	0.105	0.086	-0.04	0.062	0.901	-1.78	7.268
BMZI_6	-0	**0.872**	0.009	0.126	0.041	0.131	0.055	0.845	-1.46	5.422
BMZI_7	0.041	**0.878**	0.002	0.059	0.112	0.012	0.091	0.832	-1.51	6.261
BMZI_8	0.048	**0.715**	0.04	0.195	0.087	-0.02	0.372	0.871	-1.9	8.096
BMZI_9	0.03	0.477	0.047	0.221	0.015	0.022	**0.654**	0.845	-1.48	5.101
BMZI_10	0.14	0.175	0.04	0.104	0.071	0.121	**0.851**	0.893	-0.9	2.925
BMZI_11	0.289	0.1	0.215	0.044	0.262	**0.737**	0.119	0.895	-0.21	2.074
BMZI_12	0.333	-0.01	0.12	0.046	0.137	**0.830**	0.051	0.875	0.433	2.247
BMZI_13	0.256	0.211	0.104	0.18	**0.534**	0.015	0.206	0.942	-0.91	3.37
BMZI_14	0.247	0.1	0.147	-0.001	**0.859**	0.19	-0.02	0.794	-0.23	1.966
BMZI_15	0.286	0.076	0.201	0.036	**0.830**	0.177	-0.01	0.819	-0.14	1.961
BMZI_16	**0.756**	-0.01	0.095	0.072	0.292	0.255	0.077	0.922	0.202	2.009
BMZI_17	**0.826**	0.022	0.132	0.066	0.239	0.189	0.05	0.911	0.246	1.991
BMZI_18	**0.892**	0.037	0.104	0.008	0.127	0.133	0.015	0.928	0.276	1.982
BMZI_19	**0.930**	-0.004	0.076	0.039	0.101	0.078	0.054	0.835	0.455	2.148
BMZI_20	**0.912**	0.012	0.072	0.028	0.102	0.062	0.058	0.862	0.442	2.135
BMZI_21	0.049	0.065	0.04	**0.892**	0.047	0.001	0.117	0.719	-1.04	3.125
BMZI_22	0.064	0.175	-0.001	**0.855**	0.072	-0.05	0.1	0.785	-1.48	5.149
BMZI_23	0.033	0.175	-0.01	**0.806**	0.021	0.122	0.042	0.837	-0.9	2.965

[1] Overall Kaiser–Meyer–Olkin (KMO) statistic = 0.862. Bartlett's test of sphericity (χ^2 = 8965.67, df = 253, p <0.001). The correlation matrix showed correlations ranging from -0.013 to 0.905.

Table S2: Confirmatory factor analysis results for standardized factor loadings and reliability estimates for the seven-factor Ar-BMZI model

Factor	Item	Factor loadings		Reliability		
		Std.All	P.value	alpha	McDonald omega	AVE
Aesthetics	BMZI_14	0.915		0.904	0.902	0.885
	BMZI_15	0.954	<0.001			
Competition/Performance	BMZI_11	0.803		0.677	0.786	0.555
	BMZI_12	0.734	<0.001			
	BMZI_13	0.656	<0.001			
Contact	BMZI_16	0.876		0.942	0.979	0.894
	BMZI_17	0.908	<0.001			
	BMZI_18	0.901	<0.001			
	BMZI_19	0.977	<0.001			
	BMZI_20	0.947	<0.001			
Distraction	BMZI_1	0.84		0.873	0.879	0.715
	BMZI_2	0.814	<0.001			
	BMZI_3	0.893	<0.001			
	BMZI_4	0.797	<0.001			
Figure/Appearance	BMZI_21	0.881		0.836	0.855	0.774
	BMZI_22	0.915	<0.001			
	BMZI_23	0.789	<0.001			
Fitness	BMZI_5	0.865		0.860	0.872	0.817
	BMZI_6	0.909	<0.001			
	BMZI_7	0.923	<0.001			
Health	BMZI_10	0.641	<0.001	0.717	0.742	0.792
	BMZI_8	0.948				
	BMZI_9	0.802	<0.001			

Fit indicators: $\chi^2(209) = 1170.84$, $p < 0.001$, CFI = 0.995, TLI = 0.994, RMSEA = 0.072 (90% CI, 0.067 to 0.077), and SRMR = 0.061

Table S3: Standardized correlations between Ar-BMZI factors (seven factors).

**Factor1**	**Factor2**	**Std.All**	**P.value**
Distraction	Fitness	0.160	0.001
	Health	0.173	0.002
	Competition_Performance	0.438	< 0.001
	Aesthetics	0.410	< 0.001
	Contact	0.292	< 0.001
	Figure_Appearance	0.064	0.163
Fitness	Health	0.824	< 0.001
	Competition_Performance	0.319	< 0.001
	Aesthetics	0.271	< 0.001
	Contact	0.060	0.191
	Figure_Appearance	0.389	< 0.001
Health	Competition_Performance	0.406	< 0.001
	Aesthetics	0.246	< 0.001
	Contact	0.207	< 0.001
	Figure_Appearance	0.493	< 0.001
Competition_Performance	Aesthetics	0.686	< 0.001
	Contact	0.685	< 0.001
	Figure_Appearance	0.258	< 0.001
Aesthetics	Contact	0.579	< 0.001
	Figure_Appearance	0.143	0.002
Contact	Figure_Appearance	0.125	0.004

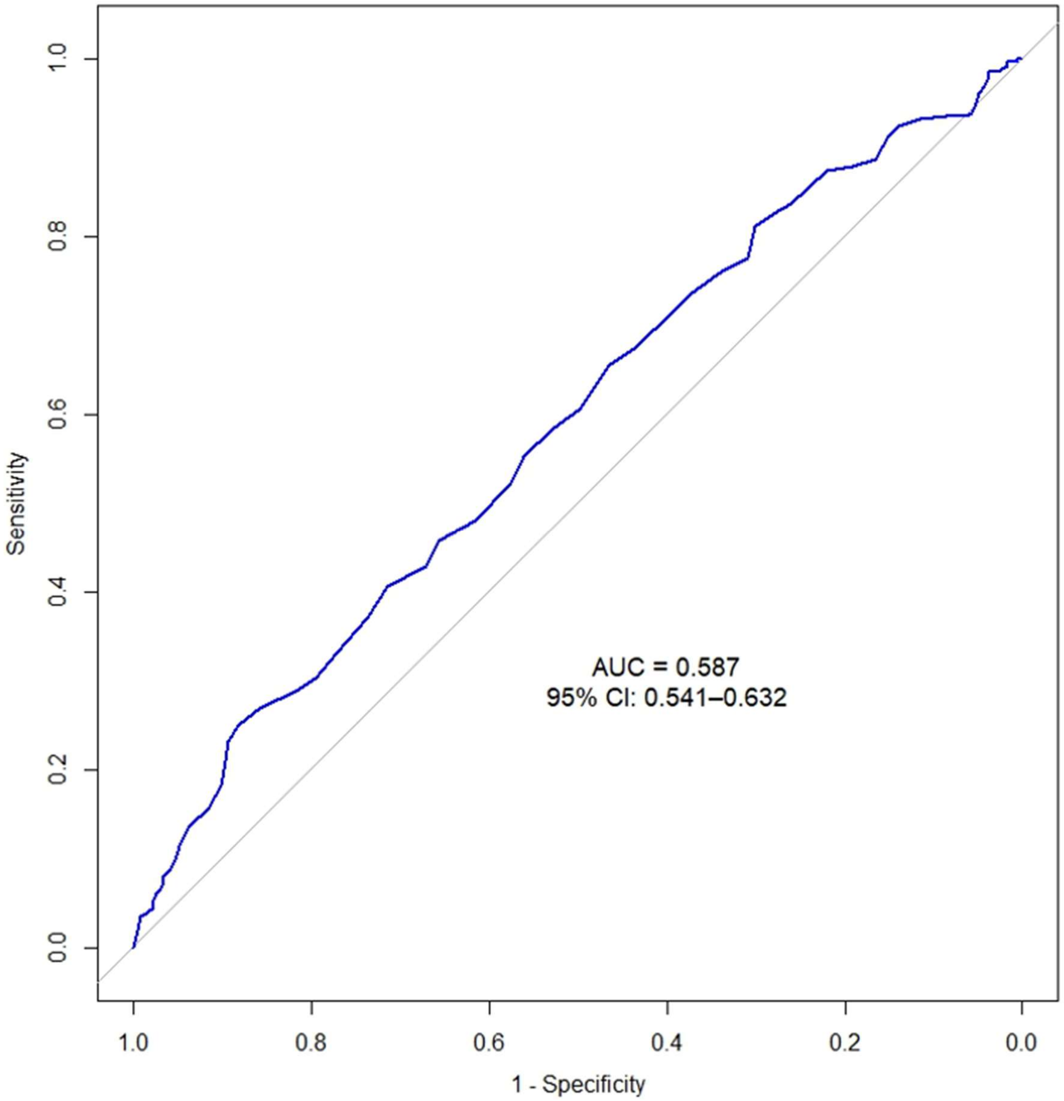

Figure S1: For the Exploratory ROC curve analysis, participants in the action and maintenance stages of change were classified as the positive group, whereas those in pre-contemplation, contemplation, and preparation were classified as the negative group. The BMZI total score showed an AUC of 0.591 (SE = 0.022, 95% CI: 0.548–0.634, $p < 0.001$), indicating a statistically significant yet weak discriminative ability. The optimal cutoff point of 92.5 provided a sensitivity of 25.0% and specificity of 88.8%, corresponding to a Youden's Index of 0.138.
