# Supplementary material for: Cultural Adaptation and Psychometric Evaluation of the Arabic Bernese Motive and Goal Inventory (Ar-BMZI) in Physical Health: A General Population Study Among Adults
Source: Healthcare (Basel). 2026 Jun 17;14(12):1750. doi: 10.3390/healthcare14121750 (PMC13299709; doi:10.3390/healthcare14121750)
Supplement: Supplementary file 1 [file healthcare-14-01750-s001.zip › healthcare-4282399-supplementary file S1.pdf]

قائمة بِرْن للدّوافع والأهداف لممارسة التمارين الرياضية

لماذا تمارس التمارين الرياضية؟/ ما الأسباب التي يمكن أن تدفعك لممارسة التمارين الرياضية؟

البند	السؤال	لا أتفق بشدة	لا أتفق	محايد	أتفق	أتفق بشدة
إلهاء/تنفيس_1	لتقليل الغضب والتوتر.					
إلهاء/تنفيس_2	لألهي نفسي عن المشاكل الأخرى.					
إلهاء/تنفيس_3	لتقليل الضغوطات.					
إلهاء/تنفيس_4	لتنظيم أفكاري.					
لياقة بدنية_1	للحفاظ على لياقتي البدنية.					
لياقة بدنية_2	أمارس التمارين الرياضية بالأساس لأكونَ لائقًا بدنيًا.					
لياقة بدنية_3	بالأساس لتحسين لياقتي البدنية.					
صحة_1	بالأساس لتحسين صحتي.					
صحة_2	السبب الرئيسي هو لتحسين حالتي الصحية.					
صحة_3	لمواجهة المشكلات المؤثرة في الصحة البدنية (مثل المشاكل الصحية المزمنه ومشاكل العضلات والمفاصل).					
منافسة/أداء_1	لأن المنافسة تدفعني للنمو وتُخرِجُ أفضلَ ما لديّ.					
منافسة/أداء_2	للتنافس مع الآخرين.					
منافسة/أداء_3	لتحقيق أهداف التمارين الرياضية (مثل المشي ٣٠ دقيقة يوميًا لمدة خمسة أيام في الأسبوع).					
الجمال الحركي_1	للاستمتاع بحركات الجسم الجميلة أثناء التمارين الرياضية					
الجمال الحركي_2	لأن ممارسة التمارين الرياضية تُتيح لي فرصة القيام بحركاتٍ جميلة					
تفاعل اجتماعي_1	لأكون اجتماعيًا مع الآخرين (مثل التفاعل والدردشة خلال صف تمارين جماعي)					
تفاعل اجتماعي_2	للقيام بنشاطٍ ضمن مجموعة.					
تفاعل اجتماعي_3	للالتقاء بالأصدقاء والمعارف.					
تفاعل اجتماعي_4	للتعرّف على أشخاص.					
تفاعل اجتماعي_5	لتكوين صداقات جديدة من خلال ممارسة التمارين الرياضية.					
القوام/المظهر_1	لإنقاص وزني.					
القوام/المظهر_2	للتحكم وضبط وزني.					
القوام/المظهر_3	بسبب شكل جسمي.					

يُتبع كل نطاق بعدد (مثلًا، لياقة_1، لياقة_2، لياقة_3) يشير إلى ترتيبه في المقياس. على سبيل المثال: لياقة_2 = العنصر الثاني في نطاق اللياقة.

للاستجابة للمقياس: كل بند مصحوب بمقياس من 5 درجات (1= "لا أتفق بشدة" إلى 5= "أتفق بشدة")

Bernese Motive and Goal Inventory (BMZI)

Item	Why do you exercise?/Why would you exercise?
discat1	To reduce anger and tension.
discat2	To distract myself from other problems.
discat3	To reduce stress.
discat4	To organize my thoughts.
fit1	To keep myself in good physical shape.
fit2	Primarily to be fit.
fit3	Primarily to do something for my physical fitness.
heal1	Primarily for health reasons.
heal2	Primarily to improve my state of health
heal3	To work against physical health problems.
comper1	Because I thrive on competition.
comper2	To compete with others
comper3	To achieve my exercise goals.
aes1	For enjoyment of beautiful movements in exercise.
aes2	Because exercise offers me the possibility for beautiful movements.
con1	To be social with others.
con2	To do something in a group.
con3	To meet friends and acquaintances.
con4	To get to know people.
con5	To make new friends through exercise.
figapp1	To lose weight.
figapp2	To regulate my weight.
figapp3	Because of my body shape.

Code Prefix	Motive Domain
**discat**	Distraction/Catharsis
**fit**	Fitness
**heal**	Health
**comper**	Competition/Performance
**aes**	Aesthetics (beauty of movement)
**con**	Contact (social interaction)
**figapp**	Figure/Appearance

Each is followed by a number (e.g., fit1, fit2, fit3) indicating its order in the scale. So, for example, fit2 = the second item under the **Fitness** domain. For Response Scale: Each item is accompanied by a 5-point response scale (1 = “I strongly disagree” to 5 = “I strongly agree”)
