# Supplementary material for: Cultural Adaptation and Psychometric Evaluation of the Arabic Bernese Motive and Goal Inventory (Ar-BMZI) in Physical Health: A General Population Study Among Adults
Source: Healthcare (Basel). 2026 Jun 17;14(12):1750. doi: 10.3390/healthcare14121750 (PMC13299709; doi:10.3390/healthcare14121750)
Supplement: Supplementary file 1 [file healthcare-14-01750-s001.zip › healthcare-4282399-supplementary file S3.pdf]

Checklist for Reporting Results of Internet E-Surveys (CHERRIES)

Checklist Item	***Explanation***	***Page Number***
Describe survey design	Cross-sectional web-based study targeting native Arabic-speaking adults residing in Saudi Arabia. Convenience sampling was used via social media platforms and community networks. The study was open to all eligible visitors who accessed the survey link.	p.2-3
IRB approval	Ethical approval was obtained from the Institutional Review Board (IRB) of King Saud University (No. E-25-10051; 25/0657/IRB), granted on August 26, 2025.	p.4
Informed consent	The first page of the online survey presented a clear description of the study purpose, procedures, potential benefits, and voluntary nature of participation. Participants were required to actively click an 'I agree' button to proceed, constituting explicit electronic informed consent. The survey length was implicitly indicated by its structure. Data were stored on password-protected servers accessible only to the research team.	p.4
Data protection	No personally identifiable information (such as name, national ID, or IP address) was collected. All data were stored on password-protected servers accessible only to the research team.	p.4
Development and testing	The survey was developed and administered via the SurveyMonkey platform (www.surveymonkey.com). The Ar-BMZI underwent a rigorous forward-backward translation process with expert review and face validation (n=27) before fielding. Technical and usability aspects of the SurveyMonkey platform are established and well-tested.	p.2-4
Open survey versus closed survey	Open survey: the survey link was distributed publicly via social media platforms and community networks, making it accessible to all eligible visitors without a password or pre-registration requirement.	p.2-3
Contact mode	Participants were contacted and recruited entirely online via social media platforms and community networks. No offline contact was used.	p.2-3
Advertising the survey	The survey was announced via social media platforms (e.g., Twitter/X, WhatsApp, and similar platforms) and community networks in Saudi Arabia, targeting native Arabic-speaking adults. The specific wording of announcements is not provided in the manuscript.	p.2-3
Web/E-mail	Web-based survey posted on the SurveyMonkey platform (www.surveymonkey.com). Responses were captured automatically and electronically by the platform.	p.2-3

Checklist for Reporting Results of Internet E-Surveys (CHERRIES)

Context	The survey was hosted on SurveyMonkey and distributed through social media and community networks in Saudi Arabia. This platform is general-purpose and not affiliated with any health advocacy group; however, social media recruitment may have pre-selected a more digitally engaged, younger, and more educated population.	p.2-3
Mandatory/voluntary	Voluntary. Participation was entirely optional; participants were informed they could close the browser at any time without submitting partial responses, with no penalty for non-completion.	p.4
Incentives	No incentives (monetary or non-monetary) were offered to participants.	N/A
Time/Date	Data were collected between September 2025 and October 2025.	p.2-3

Randomization of items or questionnaires	N/A	N/A
Adaptive questioning	N/A	N/A
Number of Items	The survey included: sociodemographic items (4 variables: age, gender, education, monthly income), a stage of behavior change item, 23 Ar-BMZI items, 28 SMS items, and 12 SF-12 items — totaling approximately 68+ items across the full questionnaire.	p.2-3
Number of screens (pages)	N/A	N/A
Completeness check	Participants who did not complete the survey were excluded from analysis, implying that incomplete responses were not retained. Specific technical mechanisms (e.g., JavaScript-based completeness checks) used by SurveyMonkey are not described.	p.2-3
Review step	N/A	N/A

Checklist for Reporting Results of Internet E-Surveys (CHERRIES)

Unique site visitor	N/A	N/A
View rate (Ratio of unique survey visitors/unique site visitors)	N/A	N/A
Participation rate (Ratio of unique visitors who agreed to participate/unique first survey page visitors)	N/A	N/A
Completion rate (Ratio of users who finished the survey/users who	Not explicitly reported as a ratio. The manuscript states that those who did not complete the survey were excluded, and a final sample of 680 was included. The total number who began but did not complete the survey is not provided.	p.2-3
agreed to participate)	Not explicitly reported as a ratio. The manuscript states that those who did not complete the survey were excluded, and a final sample of 680 was included. The total number who began but did not complete the survey is not provided.	p.2-3
Cookies used	N/A	N/A
IP check	N/A	N/A

Checklist for Reporting Results of Internet E-Surveys (CHERRIES)

Log file analysis	N/A	N/A
Registration	N/A	N/A
Handling of incomplete questionnaires	Only completed questionnaires were analyzed. Participants who did not complete the survey were excluded.	p.2-3
Questionnaires submitted with an atypical timestamp	N/A	N/A
Statistical correction	N/A	N/A

This checklist has been modified from Eysenbach G. Improving the quality of Web surveys: the Checklist for Reporting Results of Internet E-Surveys (CHERRIES). J Med Internet Res. 2004 Sep 29;6(3):e34 [erratum in J Med Internet Res. 2012; 14(1): e8.]. Article available at https://www.jmir.org/2004/3/e34/; erratum available https://www.jmir.org/2012/1/e8/. Copyright ©Gunther Eysenbach. Originally published in the Journal of Medical Internet Research, 29.9.2004 and 04.01.2012.

This is an open-access article distributed under the terms of the Creative Commons Attribution License (https://creativecommons.org/licenses/by/2.0/), which permits unrestricted use, distribution, and reproduction in any medium, provided the original work, first published in the Journal of Medical Internet Research, is properly cited.
